# Efficacy and safety of novel pulsed field ablation (PFA) technique for atrial fibrillation: A systematic review and meta‐analysis

**DOI:** 10.1002/hsr2.1079

**Published:** 2023-01-19

**Authors:** Nour Shaheen, Ahmed Shaheen, Abdelraouf Ramadan, Abdulqadir J. Nashwan

**Affiliations:** ^1^ Alexandria Faculty of Medicine, Alexandria University Alexandria Egypt; ^2^ Helwan Faculty of Medicine, Helwan University Cairo Egypt; ^3^ Hamad Medical Corporation Doha Qatar

**Keywords:** arrhythmia recurrence, atrial fibrillation, pulmonary vein isolation, pulsed‐field ablation

## Abstract

**Background and Aim:**

Pulse field ablation (PFA) has emerged as a safe alternative to other catheter ablation energy sources for patients. Although early results are encouraging, secondary data about outcomes are lacking. Herein, we aimed to assess the safety and efficacy of the novel technique.

**Methods:**

We searched PubMed, Ovid, Google Scholar, Web of Science, and Scopus databases and several major scientific conferences for studies reporting results regarding PFA.

**Results:**

Sixteen studies were included, reporting 485 patients with atrial fibrillation who underwent pulsed field operations. Patients averaged 60 years of age. The total duration of the procedure is 94 min. The average Fluoro procedure takes 17 min. Isolation of all pulmonary veins was 100% with a 95% confidence interval (CI) (*p* > 0.05). Overall, the recurrence rate of arrhythmia in the participants was 2.84% (95% CI) (*p* > 0.05). Complications were detected during or after the PFA procedure at a rate of 2.23% (*p* < 0.05), with 95% CI indicating the high safety of the PFA procedure

**Conclusion:**

Using pulsed‐field ablation as a new treatment for atrial fibrillation has proven safe and effective.

## 1 INTRODUCTION

The most common arrhythmia with a lifetime risk now estimated to be 37%, atrial fibrillation (AF), is associated with a variety of symptoms and increases with age.^1^ In patients with paroxysmal and nonparoxysmal atrial fibrillation, catheter ablation is an important treatment option.^2^ Since the initial description by Haissaguerre et al. of ectopic foci in pulmonary veins responsible for the initiation of AF, pulmonary vein isolation (PVI) has been the cornerstone of any AF ablation technique.^3^

The superior vena cava, coronary sinus, left atrial (LA) free wall, crista terminalis, and ligament of Marshall have been identified as nonpulmonary vein (PV triggers).^4^, ^5^ Over the past two decades, various ablation strategies have evolved and have been used as a stand‐alone or complement to PVI isolation. These include linear lesions, ablation involving complex fractionated atrial electrograms, rotor ablation, posterior wall isolation, and ablation of trigger sites and ganglia‐plexi (GPs). Until recently almost all ablation techniques are based on thermal effect.^6^ The thermal method induces coagulation necrosis, oedema, intramural hemorrhage, and microvascular damage. Over time this leads to reparative fibrosis leading to possible findings like impaired LA function, stiff LA syndrome, and PV stenosis.^7^ An emerging nonthermal ablative modality, pulsed‐field ablation involves applying high‐voltage, ultra‐short pulses to target tissue.^8^

The objective of the systematic review and meta‐analysis is to determine the efficacy and safety of pulse field ablation (PFA) ablation strategies.

## 2 DATA SOURCES AND SEARCH STRATEGY

**Figure 1:**
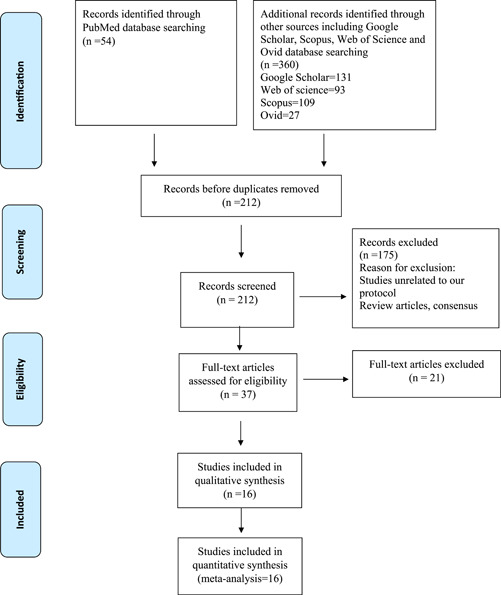
PRISMA Flow diagram of included studies. PRISMA, Preferred Reporting Items for Systematic Reviews and Meta‐Analyses.

PRISMA (Preferred Reporting Items for Systematic Reviews and Meta‐Analyses) guidelines were followed for the reporting of the study. (Figure 1). The electronic searches were conducted independently by two investigators using the same method in PubMed, Ovid, Google Scholar, Web of Science, and Scopus databases using the keywords “Pulsed‐field ablation,” “PFA,” “PF ablation,” and “atrial fibrillation.” Full‐published papers and conference abstracts were available as data sources. We further screened the bibliographies of relevant review articles to support our electronic searches.

### 2.1 Inclusion criteria

(a) Adults (≥18 years old) who have paroxysmal or nonparoxysmal atrial fibrillation, underwent (b) PFA ablation with (c) Different eligible catheter ablation approaches versus a control (and including d) Efficacy (arrhythmia‐free survival) and safety (periprocedural, postprocedural complications), (e) clinical trials/observational studies.

### 2.2 Exclusion criteria

Ablation, surgical ablation, and rate control via atrioventricular node ablation in patients with prior ablation.

### 2.3 Literature screening

The articles were electronically downloaded into reference management software (Mendeley Desktop) and duplicate articles were automatically or manually excluded. Two individual investigators screened the remaining articles according to predefined criteria. The full‐text versions of potentially relevant articles were made available and again screened by two investigators according to predefined criteria. The third investigator determined whether there were differences.

### 2.4 Data extraction

We extracted data representing the efficacy and safety of pulsed‐field ablation, including procedure outcomes (procedure ablation time, procedure‐related adverse events [complications], PVI and arrhythmia recurrence). Furthermore, baseline characteristics of patients and study characteristics were extracted.

### 2.5 Quality assessment of the included studies

Based on the Newcastle‐Ottawa Scale, the quality and reporting of the studies were evaluated. After dividing the studies into three categories, points were given to high‐quality studies (6−9 points); satisfactory‐quality studies (3−5 points); and unsatisfactory‐quality studies (0−2 points) (the Supporting Information file).^9^

### 2.6 Statistical analysis

We used the dmetar package, meta and meta for R version 4.3 pooling effect sizes and for the visual representation of the results. Means and proportions were pooled using the random effect model when the heterogeneity was below 50% and the fixed effect model when the heterogeneity exceeded 50% with 95% confidence intervals (95% CI). Additionally, *I*² was used to describe the heterogeneity between the studies, which ranged from 0% to 100% (25%, 50%, and 75% representing low, moderate, and high heterogeneity, respectively). For sensitivity analysis, we identified the outlier using the brute force approach and used the influence analysis as well.

## 3 RESULTS

Based on database searches and other resources (conference proceedings), 414 entries were identified, of which 202 were excluded as duplicates. Of the 212 publications that qualified for abstract review, 165 were excluded primarily because they were not controlled trials, or were review articles, or did not meet our criteria. The remaining 37 publications were subjected to a full article review and 19 more publications were excluded. The characteristics and quality of the 16 included studies are summarized in (Supporting Information: Tables 1 and 2). Eight studies were published as manuscripts.^8^, ^10^, ^11^, ^12^, ^13^, ^14^, ^15^, ^16^ The other eight were from data presented at conferences and published as abstracts.^17^, ^18^, ^19^, ^20^, ^21^, ^22^, ^23^, ^24^ The sample size for these studies was 485 participants (Supporting Information File).

### 3.1 Outcomes of the included studies

#### 3.1.1 Age

**Figure 2:**
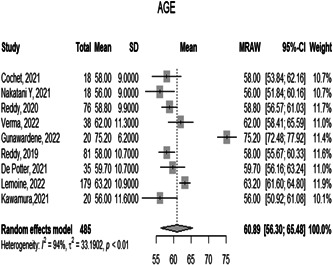
Forest plot diagram of the age of the included patients

The overall mean age of the included patients in 12 studies was 60.8919 [56.3022; 65.4815 *p* < 0.0001]. However, the observed heterogeneity among the studies was significant (*p* < 0.05, *I*² = 93.9%), (Figure 2).

#### 3.1.2 Total procedure time

**Figure 3:**
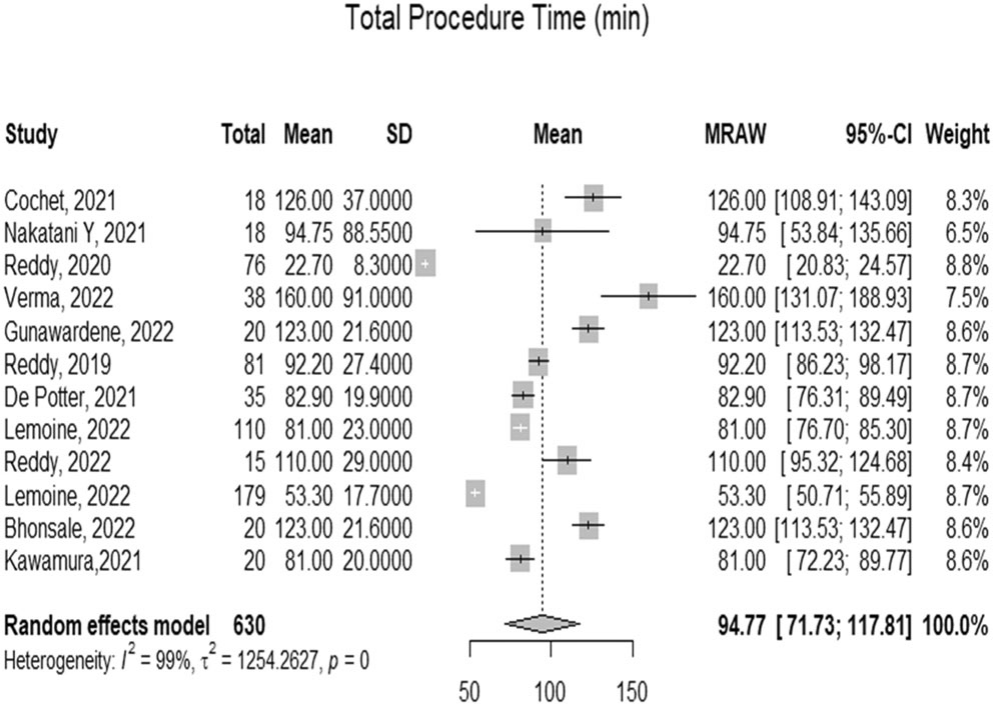
Forest plot diagram of the total procedure time (min)

The overall mean of the total procedure time of the 12 included studies is 94.77 [71.73; 117.81 *p* = 0.0021]. However, the observed heterogeneity among the studies was significant (*p* < 0.05, *I*² = 799.5%), (Figure 3).

#### 3.1.3 Fluoro time

**Figure 4:**
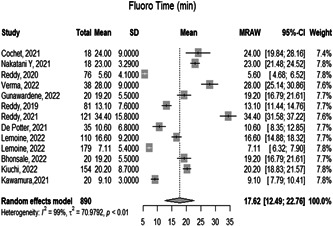
Forest plot diagram of the fluro time (min)

The overall mean time of Fluoro in the procedure in 13 studies is 17.6250 [12.4864; 22.7635 with 95% CI *p* < 0.0001]. However, the observed heterogeneity among the studies was significant (*p* < 0.05, *I*² = 99%), (Figure 4).

#### 3.1.4 Successful PVI

**Figure 5:**
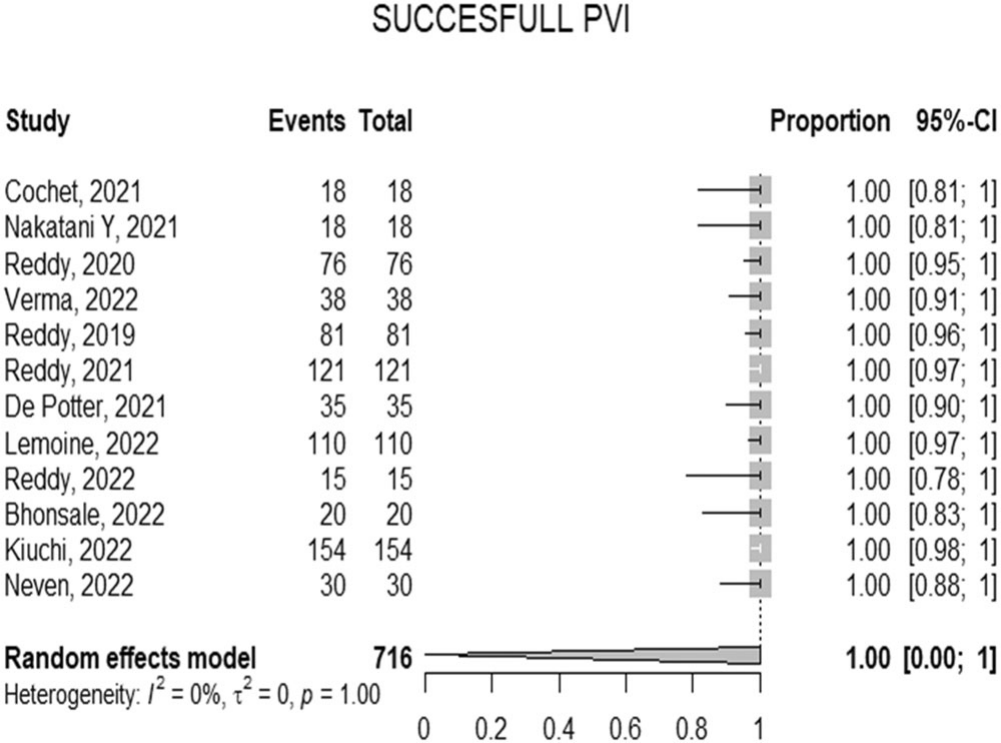
Forest plot diagram of the successful PVI. PVI, pulmonary vein isolation.

The overall proportion of successful PVI in the 12 included studies during the ablation is 1.0000 [0.0000; 1.0000 with 95% CI *p* = 1.00]. The observed heterogeneity among the studies was not significant (*p* > 0.05, *I*² = 0.0%) representing very low heterogeneity, (Figure 5).

#### 3.1.5 Arrhythmia recurrence

**Figure 6:**
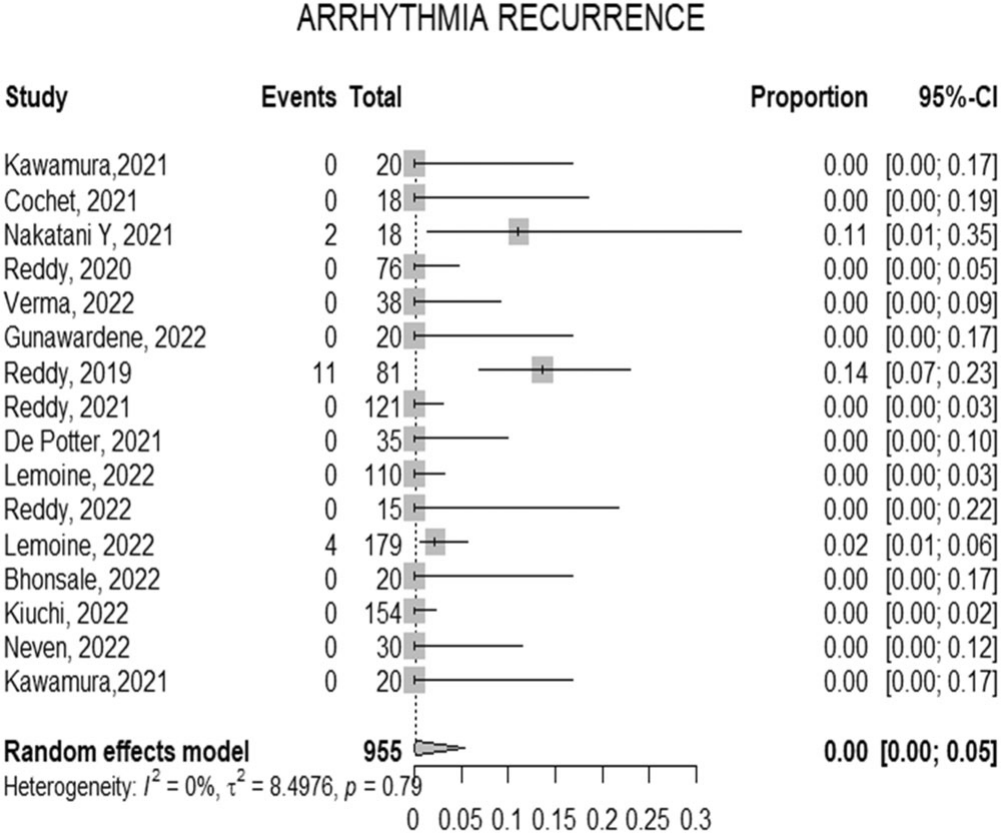
Forest plot diagram of the arrythmia recurrence

The overall proportion of arrhythmia recurrence on follow‐up of the included participants is 0.0178 [0.0111; 0.0284 *p* = 0.79] with 95% CI which is considered very little proportion and that indicates a high outcome of the PFA procedure. And the observed heterogeneity among the studies was significant (*p* < 0.05, *I*² = 0.0%), representing very low heterogeneity (Figure 6).

#### 3.1.6 Complications

**Figure 7:**
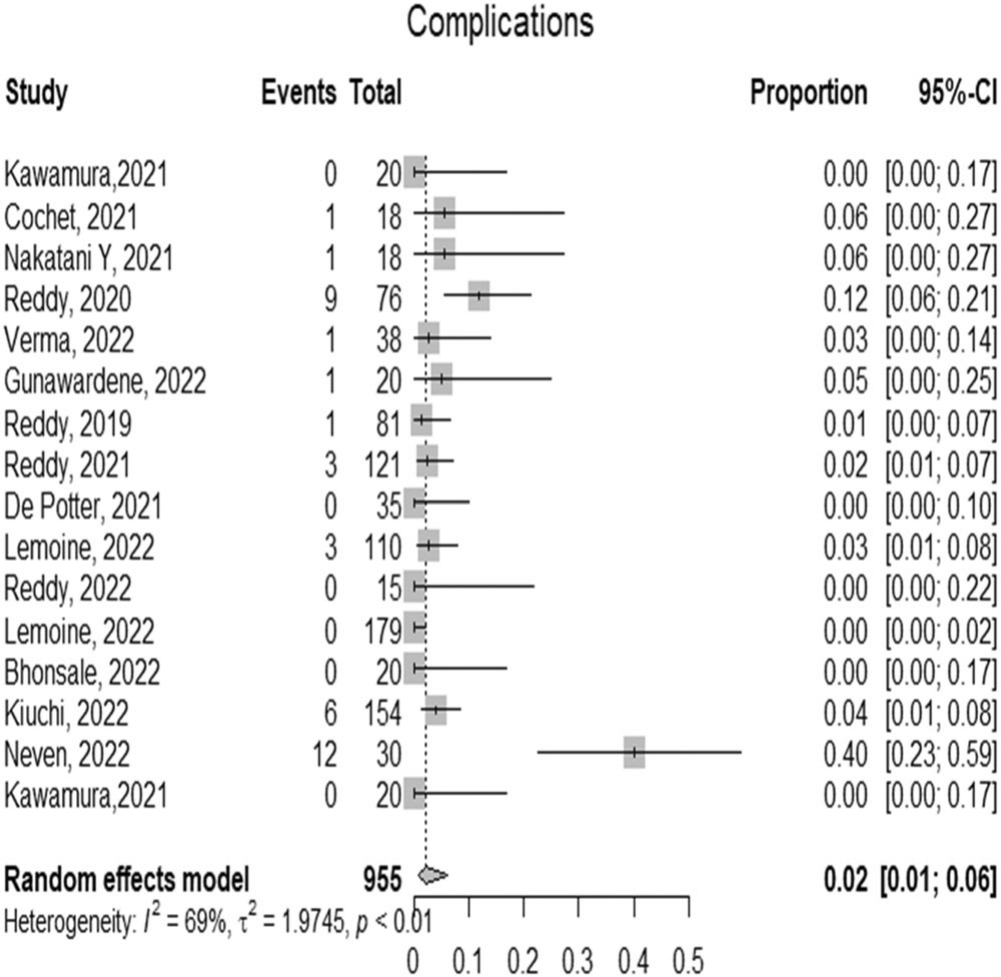
Forest plot diagram of the complications

The overall proportion of the complications either during or after the ablation is 0.0223 [0.0080; 0.0609 *p* < 0.01] with 95% CI which indicates the high safety of the PFA procedure. And the observed heterogeneity among the studies was significant (*p* < 0.05, *I*² = 0.0%), representing very low heterogeneity (Figure 7).

#### 3.1.7 Sensitivity analysis

The results of the sensitivity analysis are shown in (the Supporting Information File). There was no significant difference in any of the clinical outcomes following serial exclusion of each trial (No outliers detected [random‐effect model]).

## 4 DISCUSSION

To our knowledge, this is the first systematic review and meta‐analysis to investigate the use of pulsed‐field ablation for the treatment of atrial fibrillation. Our study includes 16 studies with a total of 485 patients who had atrial fibrillation and underwent PFA for treatment. All included studies reported total procedure time, Fluoro time, PVI, complications, and arrhythmia recurrence.

Atrial fibrillation is generally treated through medications to control heart rate and rhythms. Ablation is generally performed if medications and other therapies are ineffective. In certain patients, it is the first choice of treatment. The most commonly performed ablation is catheter ablation, a procedure in which catheters are placed in the heart through veins or arteries. Catheter ablation generally has high success rates, >80% for paroxysmal AF and >70% for persistent AF.^25^, ^26^ Catheter ablation includes various types such as radiofrequency ablation and cryoablation. In addition, PFA is also a type of catheter ablation. In this systematic review we first compare PFA to catheter to show how PFA generally compares to all types of catheter ablation without focusing on a specific type. We then compare PFA to specific types of catheter ablation specifically radiofrequency ablation and cryoablation.

### 4.1 Pulsed‐field ablation compared to previous catheter ablation procedures

The mean time of a PFA procedure is approximately 94.7 min. Catheter ablation had similar durations with a mean duration of 96 ± 36 min. The overall mean time of the Fluoro procedure is 17.63 min, based on the findings from the 13 studies which included it, compared to 5.9 ± 5.8 min in catheter ablation patients.^27^ The proportion of successful PVI during the ablation was 100% according to the data included in 12 studies, compared to a 93% success rate with catheter ablation.^27^ In addition, PVI through catheter ablation for persistent atrial fibrillation is approximately 43%.^28^

The proportion of recurrence during the follow‐up of the included participants who underwent PFA was 1.78. According to a systematic review by Cochrane, the recurrence rate for atrial fibrillation following catheter ablation was estimated to be 20.8.^26^ In a large study known as the CABANA Trial, 1240 patients were involved. After 12 months, 12.6% of the patients had recurrent symptomatic atrial fibrillation while 36.4% of patients that underwent catheter ablation had any type of atrial fibrillation which included symptomatic and asymptomatic atrial fibrillation.^25^

In this meta‐analysis, we found that the proportion of complications was very low in the patients who underwent PFA at around 2.23% which is evidence of the procedure's high safety levels. Catheter ablation acute complication rate is approximately 2.9% which is slightly larger compared with the pulsed field ablation.^29^

### 4.2 Pulsed‐field ablation compared to radiofrequency ablation

Radiofrequency ablation is also performed to treat atrial fibrillation. In a systematic review which included seven trials with 2023 patients, analyses have shown that first‐pass PVI was 80%−90% compared to 100% in our analysis.^30^ After a follow‐up of 16 months, (High Power Short Duration) HPSD, or ablation power with a short duration of 2−10 s per site, was found to reduce the recurrence of atrial arrhythmias by 70%.^31^ Additionally, HPSD ablation time is around 116 ± 41 min. The mean fluoroscopy time was 33 ± 6 min. However, major complications and oesophagal thermal injury (ETI) were approximately 0.389% which is considered a low rate to 2.23% in PFA.^32^

### 4.3 Pulsed‐field ablation compared to cryoablation

Cryoablation had a mean Procedure time of 109.9 ± 52.9 min compared to 94.7 min in PFA. The mean fluoroscopy time was 29.6 ± 14.5 min compared to 17.63 min in this meta‐analysis. PVI success rates for cryoablation are 97.6% which is lower than the PFA (100%).^33^ The proportion of recurrence rate during the follow‐up in those who had a cryoablation procedure is between 20% and 30% which indicates that PFA has a safer outcome (1.78%).^34^

The proportion of complications was very low in the patients who underwent PFA at around 2.23% compared to 3.8% in those who had cryoablation (Table 1).^35^

**Table 1:** Summary of PFA outcomes compared to other techniques

Type of ablation	Catheter ablation	Radiofrequency ablation	Cryoablation	PFA
Mean time in minutes	96 ± 36	116 ± 41	109.9 ± 52.9	94.7
Fluoroscopy time in min	5.9 ± 5.8	33 ± 6	29.6 ± 14.5	17.625
Pulmonary vein isolation	43%	80–90%	97.6%	100%
Recurrence rate	20.8%	30%	20%−30%	1.78%
Major complications rate	2.9%	0.389%	3.8%	2.3%

### 4.4 Limitations

Although this is the first meta‐analysis about the new PFA, there are some limitations of interest to be mentioned. The included studies were not randomized and did not compare pulsed ablation to other ablations or treatments for atrial fibrillation. The other limitation is that eight (8) of the sixteen (16) included studies are abstracts represented in conferences.

## 5 CONCLUSIONS

Pulsed‐field ablation is emerging as a new ablation technique used for the management of atrial fibrillation. Due to its high success rate combined with a low risk of complications and a low risk of recurrence, it will most likely be widely used in the management of atrial fibrillation. More studies, particularly clinically randomized control trial studies, need to be performed to compare this new technique with arrhythmia medications and other techniques like catheter ablations.

## AUTHOR CONTRIBUTIONS

**Nour Shaheen**: Conceptualization; data curation; formal analysis; methodology; writing – original draft; writing – review and editing. **Ahmed Shaheen**: Conceptualization; data curation; formal analysis; methodology; writing – original draft; writing – review and editing. **Abdelraouf Ramadan**: Writing – original draft; Writing – review and editing. **Abdulqadir J. Nashwan**: Writing – original draft; writing – review and editing.

## CONFLICTS OF INTEREST

Abdulqadir J. Nashwan is an Editorial Board member of Health Science Reports and coauthor of this article. He is excluded from editorial decision‐making related to the acceptance of this article for publication in the journal. The remaining authors declares no conflicts of interest.

## TRANSPARENCY STATEMENT

The lead author (Dr Nour Shaheen) affirms that this manuscript is an honest, accurate, and transparent account of the study being reported; that no important aspects of the study have been omitted; and that any discrepancies from the study as planned (and, if relevant, registered) have been explained.

## Supporting information

Supporting information.

## Data Availability

The data was extracted from online published articles.
