# Supplementary material for: Efficacy and safety of novel pulsed field ablation (PFA) technique for atrial fibrillation: A systematic review and meta‐analysis
Source: Health Sci Rep. 2023 Jan 19;6(1):e1079. doi: 10.1002/hsr2.1079 (PMC9852677; doi:10.1002/hsr2.1079)
Supplement: Supplementary file 1 — Supporting information. [file HSR2-6-e1079-s001.docx]

Table 1: Baseline characteristics of the included studies

| First author, Year of publication | Study Design | Funding source | sex | | Number of patients | | Mean age | |
| --- | --- | --- | --- | --- | --- | --- | --- | --- |
|  |  |  | PFA | Another ablation | PFA | Other  ablation | PFA | Other  ablation |
| Kawamura,2021^1^ | Non-randomized trial | FarapulseInc. | m=15/f5 | 25 (64.1%) | 20 | thermal ablation=39 | 56.9±11.0 | 66.1 ± 9.3 |
| Cochet, 2021^2^ | Non-randomized trial | l’Agence Nationale de la Recherche (ANA) | m=15/f=5 | m=25/f=14 | 18 | thermal ablation=46 | 58 ± 9 | 59 ± 9 |
| Nakatani Y, 2021^3^ | Non-randomized trial | l’Agence Nationale de la Recherche (ANA) | m=15/f=3 | m=17 /f=6 | 18 | thermal ablation=23 | 56 ± 9 | 60 ± 8 |
| Reddy, 2020^4^ | Non-randomized trial | Affera | m=50/f=26 | NA | 76 | NA | 58.8±9.9 | NA |
| Verma, 2022^5^ | Non-randomized trial | Medtronic, Inc. | m=20/f=18 |  | 38 | NA | 62.0±11.3 | NA |
| Gunawardene, 2022^6^ | Non-randomized trial | No fund | m=6 /f=5 | m=6 /f=3 | 11 | 9 | 75.2 ± 6.2 | 64.4 ± 10.2 |
| Reddy, 2019^7^ | Non-randomized trial | Farapulse | m=60 /f=21 | NA | 81 | NA | 58.0 ± 10.7 | NA |
| Reddy, 2021^8^ | Non-randomized trial | Farapulse Inc | m=89 (73.6) \f=22 | NA | 121 | NA | 57.4 ± 10.3 | NA |
| De Potter, 2021^9^ | Non-randomized trial | inspIRE | 54.3% male | NA | 35 | NA | 59.7±10.7 | NA |
| Lemoine, 2022^10^ | Non-randomized trial | NA | 62% male | NA | 110 | NA |  | NA |
| Reddy, 2022^11^ | Non-randomized trial | NA | NA | NA | 15 | NA | 63±9 | NA |
| Neuzil, 2022^12^ | Non-randomized trial | NA | 118 M/ 61 F | NA | 179 | NA | 63,2 ±10,9 | NA |
| M. Gunawardene, 2022^13^ | Non-randomized trial | NA | NA | NA | 20 | NA |  | NA |
| (M. Gunawardene, Lemoine, 2022^14^ | Non-randomized trial | NA | NA | NA | 154 | NA |  | NA |
| Neven, 2022^15^ | Non-randomized trial | NA | 47% male | NA | 30 | NA | 63 | NA |
| Kawamura,2021^16^ | Non-randomized trial | Farapulse Inc. | Male sex 15 (75.0) | NA | 20 | NA | 56.0 ± 11.6 | NA |

Table2: demographics of the first Eight (8) included studies.

| **First author, Year of publication** |  | **Kawamura,2021** | Cochet, 2021 | Nakatani Y, 2021 | Reddy, 2020 | Verma, 2022 | Gunawardene, 2022 | **Reddy, 2019** | Reddy, 2021 |
| --- | --- | --- | --- | --- | --- | --- | --- | --- | --- |
| **Paroxysmal atrial fibrillation** | **PFA** | NA | NA | NA | 55/76 (72%) | **35(92%)** | 7 (77.8) | NA | NA |
|  | **another ablation** | NA | NA | NA | NA |  | 0 (0.0) | NA | NA |
| **Left atrial diameter, mm** | **PFA** | 41.7 ± 5.0 | NA |  | 42.6±5.3 | 37.3± 5.8 | 45.2 ± 4.1 | **41.2 ± 5.0** | **40.5± 4.5** |
|  | **another ablation** | 41.1 ± 6.0 (n = 32) | NA | NA | NA |  | 42.1 ± 5.8 | NA | NA |
| **CAD** | **PFA** | NA | **1 (6%)** | NA | NA | NA | NA | NA | NA |
|  | **another ablation** | NA | **2 (9%)** | NA | NA | NA | NA | NA | NA |
| **Left ventricular ejection fraction, %** | **PFA** | 63.6 ± 3.7 | 62 ± 6 | 62 ± 6 | 58.1±5.8 | 59.5± 5.1 | 10 (90.9) | **63.3 ± 4.3** | **62.5± 5.7** |
|  | **another ablation** | 60.8 ± 7.5 (n = 35) | 61 ± 8 | 61 ± 8 |  |  | 5 (55.6) |  |  |
| **Antiarrhythmic drugs (%)** | **PFA** |  | **AAD class I  9 (50%)   AAD class II  8 (44%)   AAD class III  5 (28%)   No AAD  4 (22%)** | 13 (72) | Class I–IV 72/76 (95%) Class I or III 47/76 (62%) | **n=1.3± 0.5** |  | **Class I 46 Class II 45 Class III 1 No 12** |  |
|  | **another ablation** |  | **AAD class I  7 (30%)   AAD class II  9 (39%)   AAD class III  10 (43%)   No AAD  6 (26%)** | 18 (78) | NA | NA | NA | NA | NA |
| **Anticoagulants (%)** | **PFA** |  | **Warfarin  0 (0%)   NOAC  18 (100%)** | 18 | **Warfarin, n (%) = 19/76 (25%) NOAC, n (%) 50/76 (66%)** |  |  | **Warfarin 32 NOAC 35 Aspirin 2 No 12** |  |
|  | **another ablation** | NA | **Warfarin 2 (9%)   NOAC  21 (91%)** | 21 | NA | NA | NA | NA | NA |
| **Hypertension (%)** | **PFA** | NA | 4 (22%) | 4 (22) | **55/76 (72%)** | NA | NA | **50 (61.7)** | **68 (56.2)** |
|  | **another ablation** | NA | 4 (17%) | 4 (17) |  | NA | NA |  |  |
| **Diabetes (%)** | **PFA** | NA | 1 (6%) | 1 (6) | **6/76 (8%)** | NA | NA | **8 (9.9)** | **11 (9.1)** |
|  | **another ablation** | NA | 0 (0%) | 0 0 | NA | NA | NA |  |  |
| **Previous stroke/TIA (%)** | **PFA** | NA | 2 (11%) | NA | NA | NA | NA | **3 (3.7)** | **6 (5.0)** |
|  | **another ablation** | NA | 1 (4%) | NA | NA | NA | NA | NA | NA |
| **Years since atrial fibrillation diagnosis** |  | NA | NA | NA | NA | 7.2± 1.8 |  | NA | NA |
| **Mean (SD) CHA2DS2-VASc  score (ablation vs AADs)** | **PFA** | NA | NA | 0.5 [0–1] | NA | 1.9± 1.6 | 3 [2–3.5] | NA | NA |
|  | **another ablation** | NA | NA | 1 [0–1] | NA |  | 2 [1–4] | NA | NA |
| **Type of ablation** | **PFA** | **pfa=20** | **PFA** | **PFA** | NA |  |  | NA | NA |
|  | **another ablation** | **Thermal energy ablation=39** | **N=23, 16 radiofrequency, 7 cryoballoon** | **Thermal energy ablation** | NA | catheter ablation |  | NA | NA |
| **Total follow-up 1** | **PFA** | NA | NA | 9 ± 3 | NA | NA | NA | NA | NA |
|  | **another ablation** | NA | **t 3-month** | 9 ± 4 | NA | NA | NA | NA | NA |

Table 3: quality assessment of the included studies

| **Study** | **Selection** | | | | **Comparability** | | **Outcome** | | | **Total** |
| --- | --- | --- | --- | --- | --- | --- | --- | --- | --- | --- |
|  | **1** | **2** | **3** | **4** | **1** | **2** | **1** | **2** | **3** |  |
| Kawamura,2021^1^ | * | * | * | * | * | * | * | * | * | 9 |
| Cochet, 2021^2^ | * | * | * | * | * |  | * |  | * | 7 |
| Nakatani Y, 2021^3^ | * | * | * | * | * | * | * | * | * | 9 |
| Reddy, 2020^4^ | * | * | * | * | * |  | * | * | * | 8 |
| Verma, 2022^5^ | * | * | * | * | * |  | * |  | * | 7 |
| Gunawardene, 2022^6^ | * | * | * | * | * | * | * | * | * | 9 |
| Reddy, 2019^7^ | * | * | * | * | * | * | * | * | * | 9 |
| Reddy, 2021^8^ | * | * | * | * | * | * | * | * | * | 9 |
| De Potter, 2021^9^ | * |  |  | * | * |  | * |  |  | 4 |
| Lemoine, 2022^10^ | * |  |  | * | * |  | * |  |  | 4 |
| Reddy, 2022^11^ | * |  |  | * | * |  | * |  |  | 4 |
| Neuzil, 2022^12^ | * |  |  | * | * |  | * |  | * | 5 |
| M. Gunawardene, 2022^13^ | * | * |  | * | * |  | * |  |  | 5 |
| (M. Gunawardene, Lemoine, 2022^14^ |  |  |  | * | * |  |  | * | * | 4 |
| Neven, 2022^15^ | * | * |  | * | * |  | * |  |  | 5 |
| Kawamura,2021^16^ | * | * | * | * | * | * | * | * | * | 9 |

Table 4: PVI (after Sensitivity analysis)

| Analysis | g | 95%CI | p | 95%PI | I2 | 95%CI |
| --- | --- | --- | --- | --- | --- | --- |
| Infl. Cases Removed1 | 1.0000 | [0.0000; 1.0000] | =1.00 | 1.0000 [0.0000; 1.0000] | 0.0% | [0.0%; 58.3%] |

Table 5: Complications (after Sensitivity analysis)

| Analysis | g | 95%CI | p | 95%PI | I2 | 95%CI |
| --- | --- | --- | --- | --- | --- | --- |
| Infl. Cases Removed1 | 0.0223 | [0.0080; 0.0609] | <0.01 | [0.0063; 0.0543] | 0.0% | [0.0%; 55.0%] |

**Publication bias**


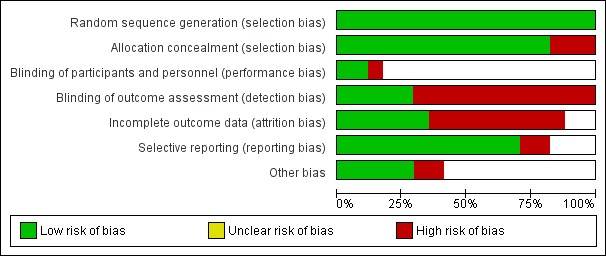


Figure 1 Risk of bias graph: review authors' judgements about each risk of bias item presented as percentages across all included studies.


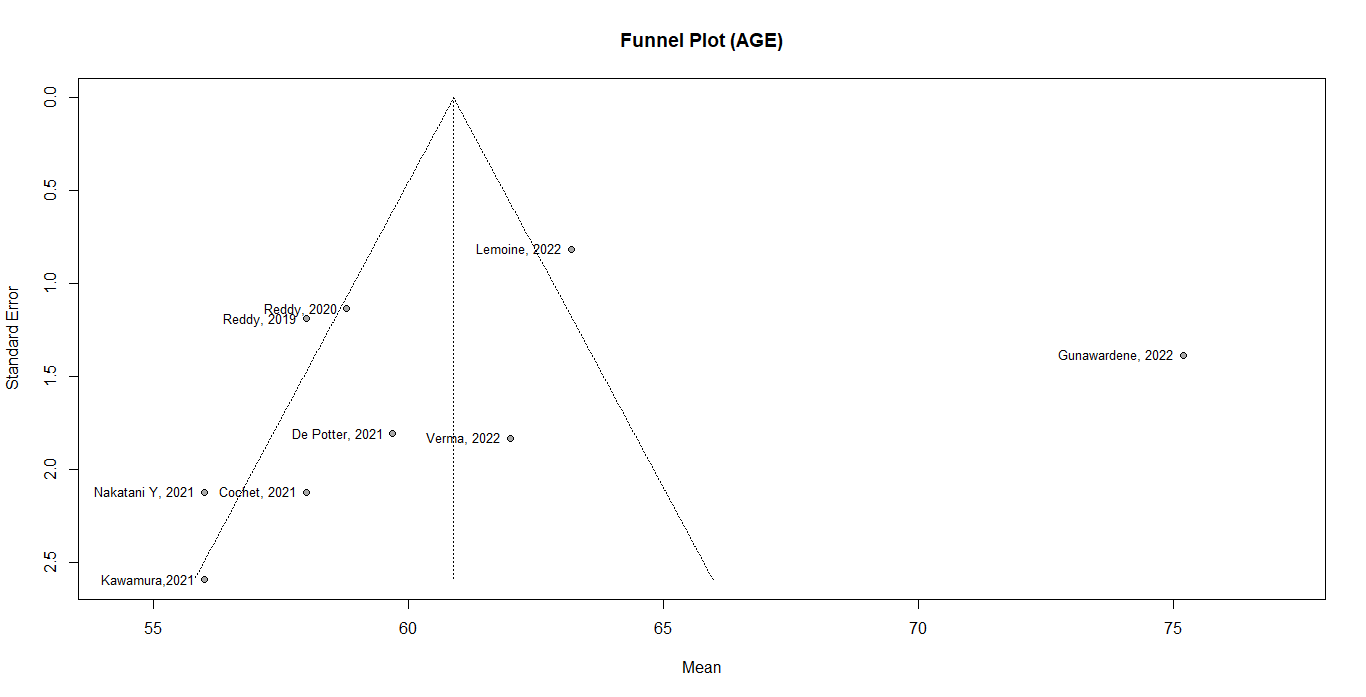


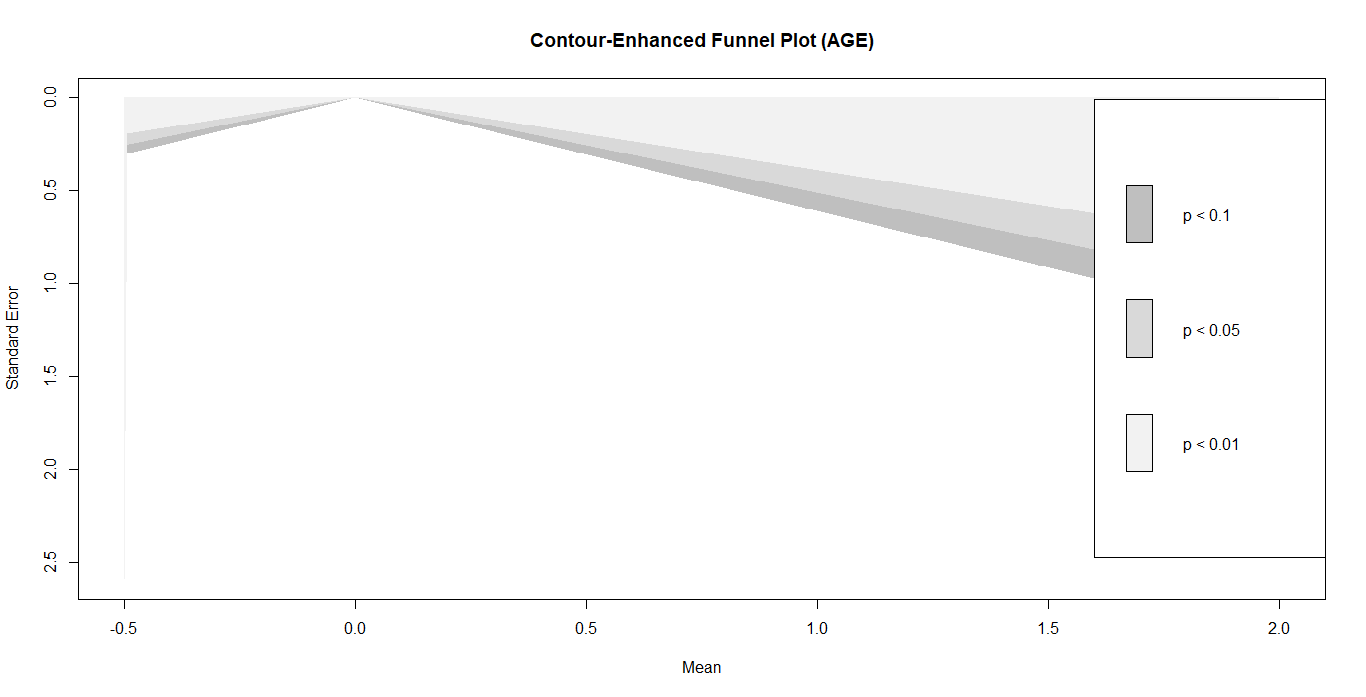


Figure 2: Funnel plot for age


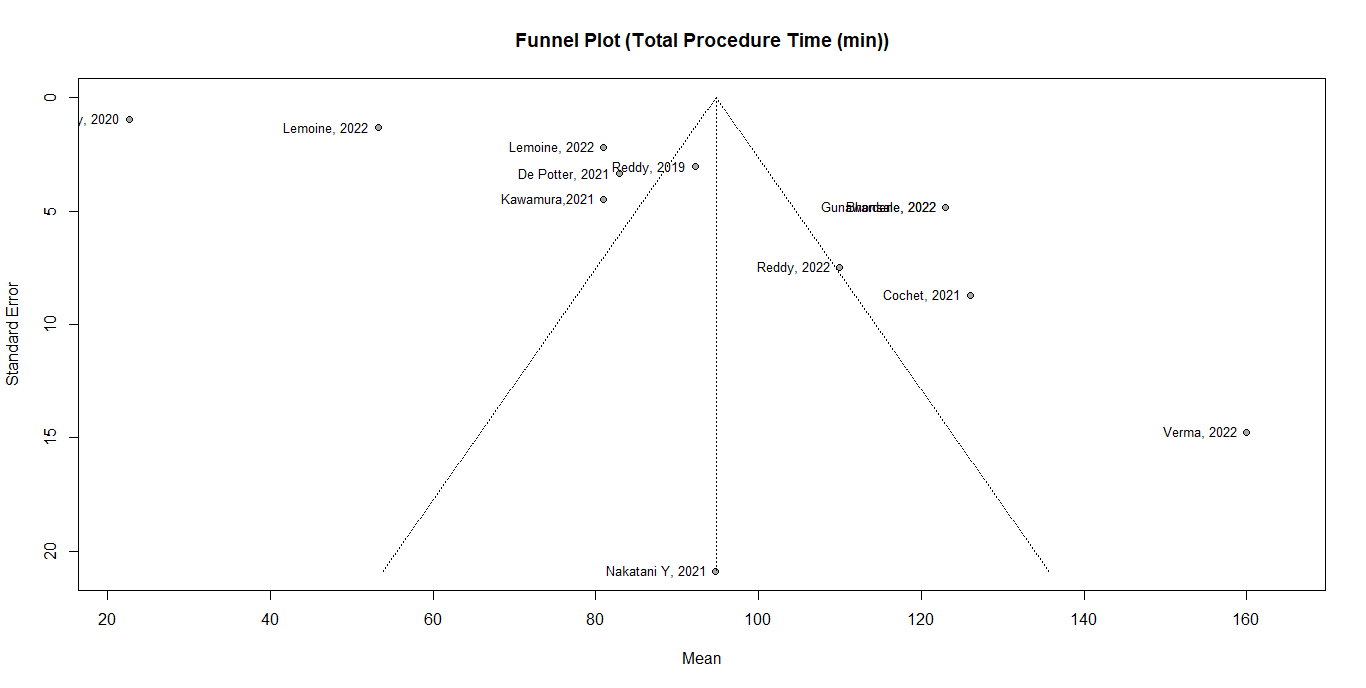


Figure 2 funnel plot for procedure time


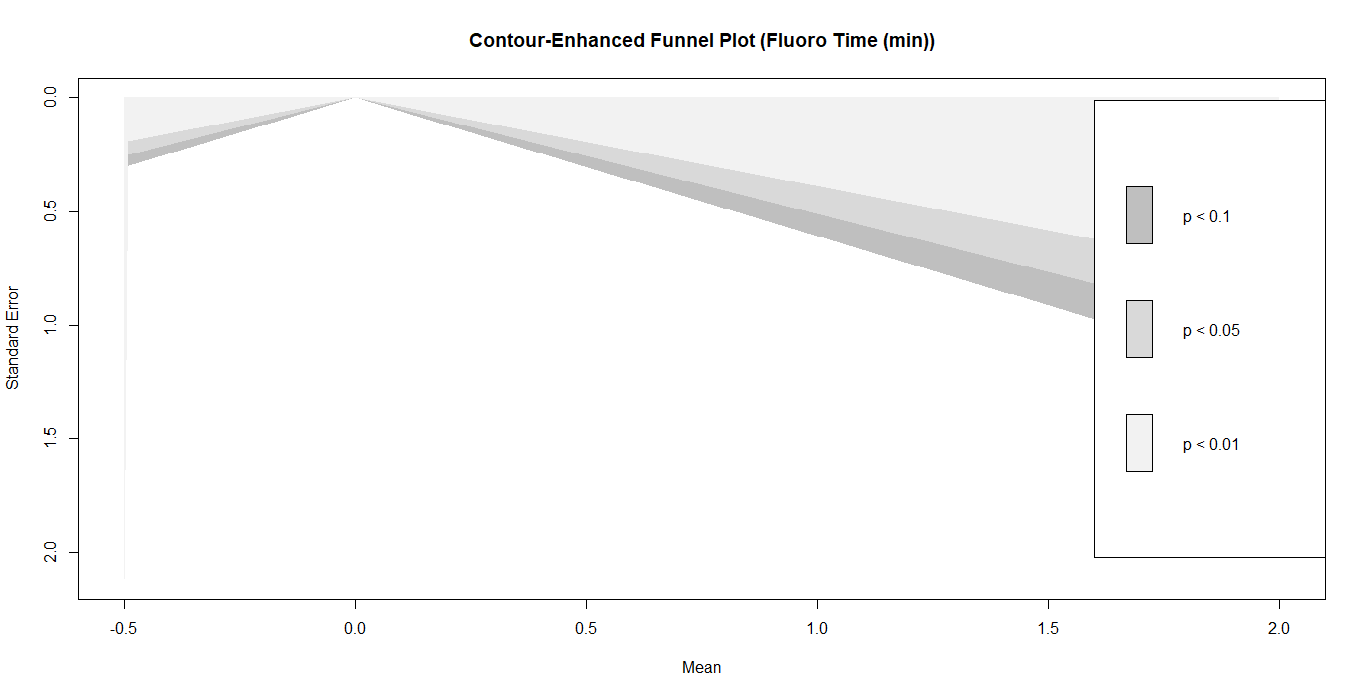


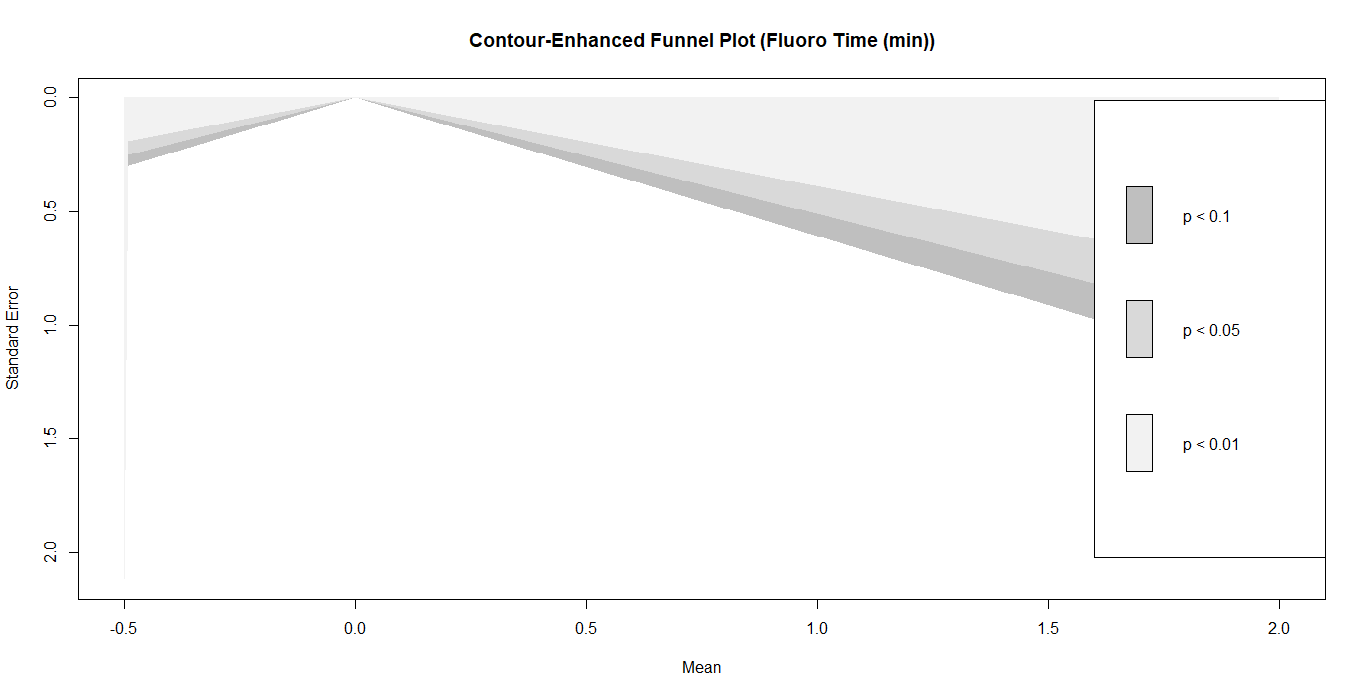


Figure 4: Funnel plot for fluro time


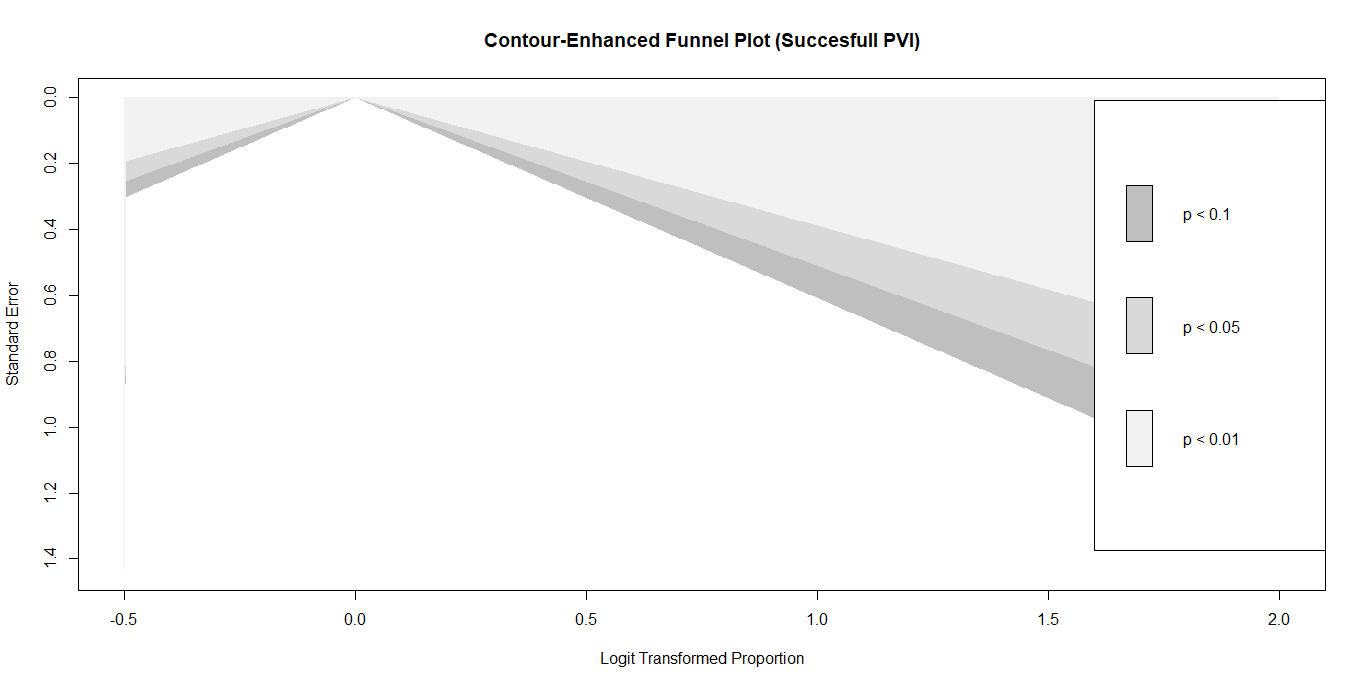


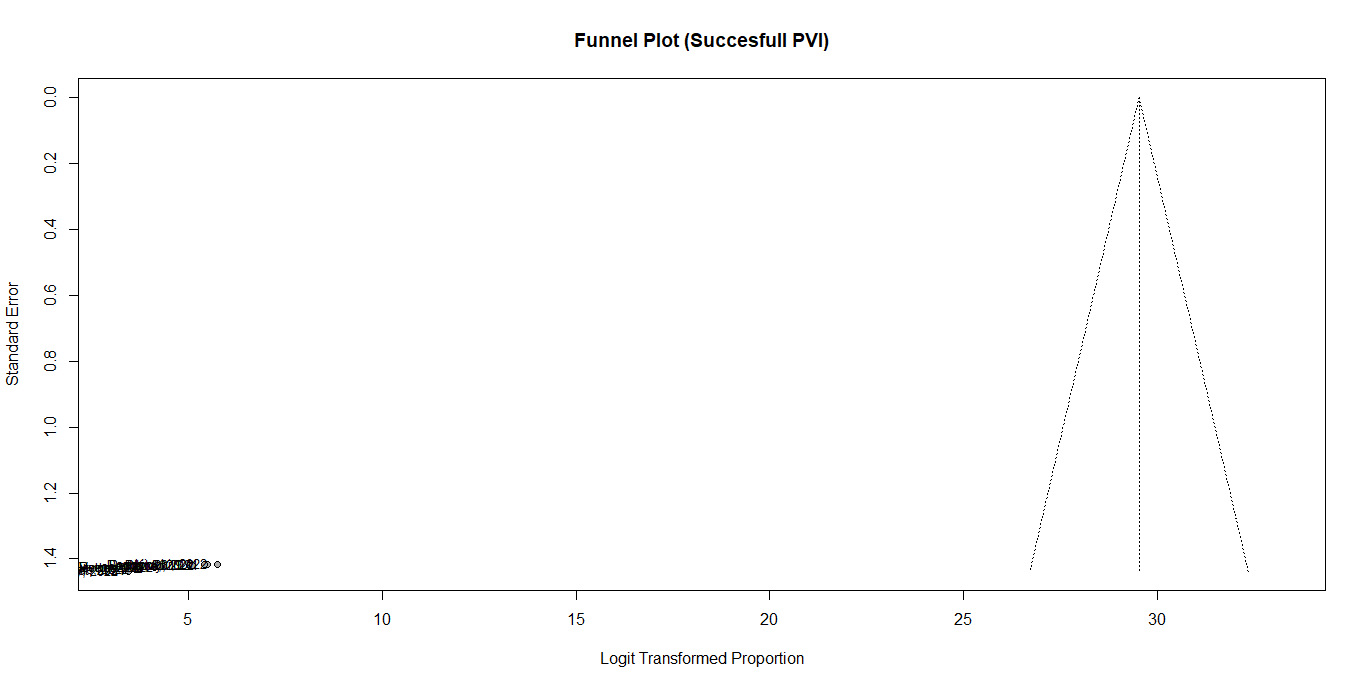


Figure 6Funnel plot for PVI


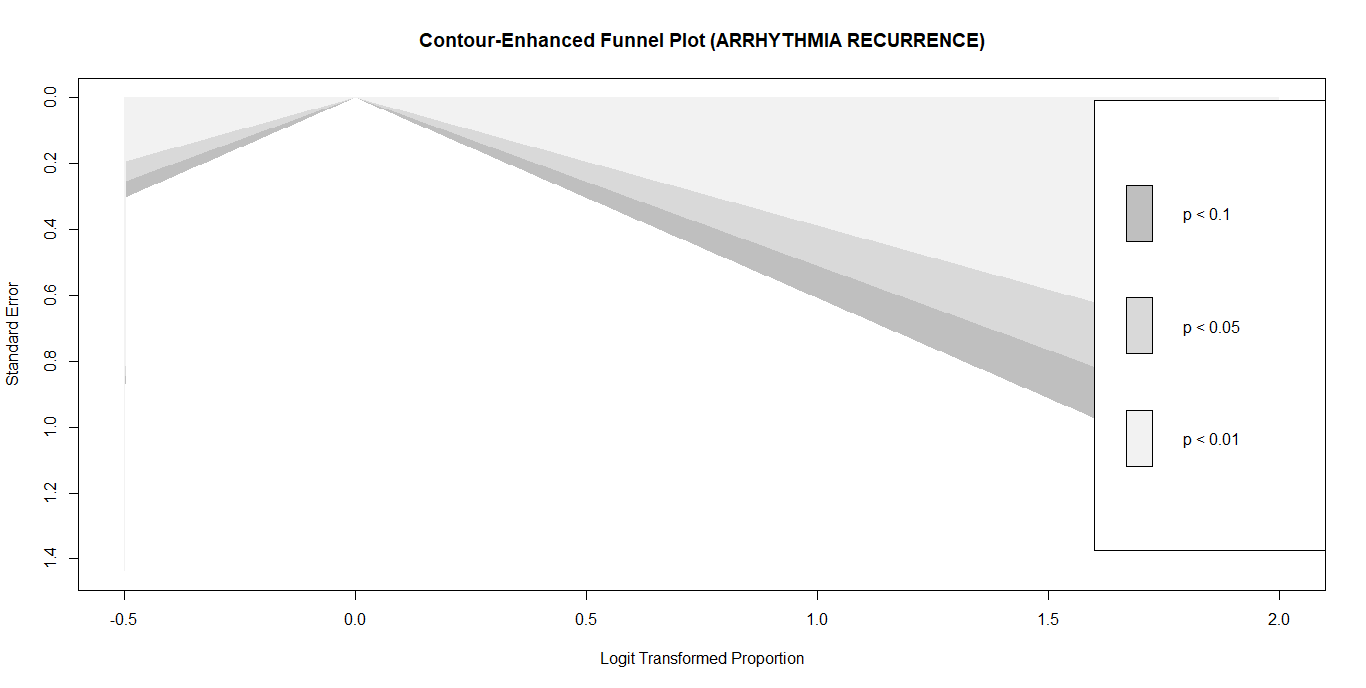

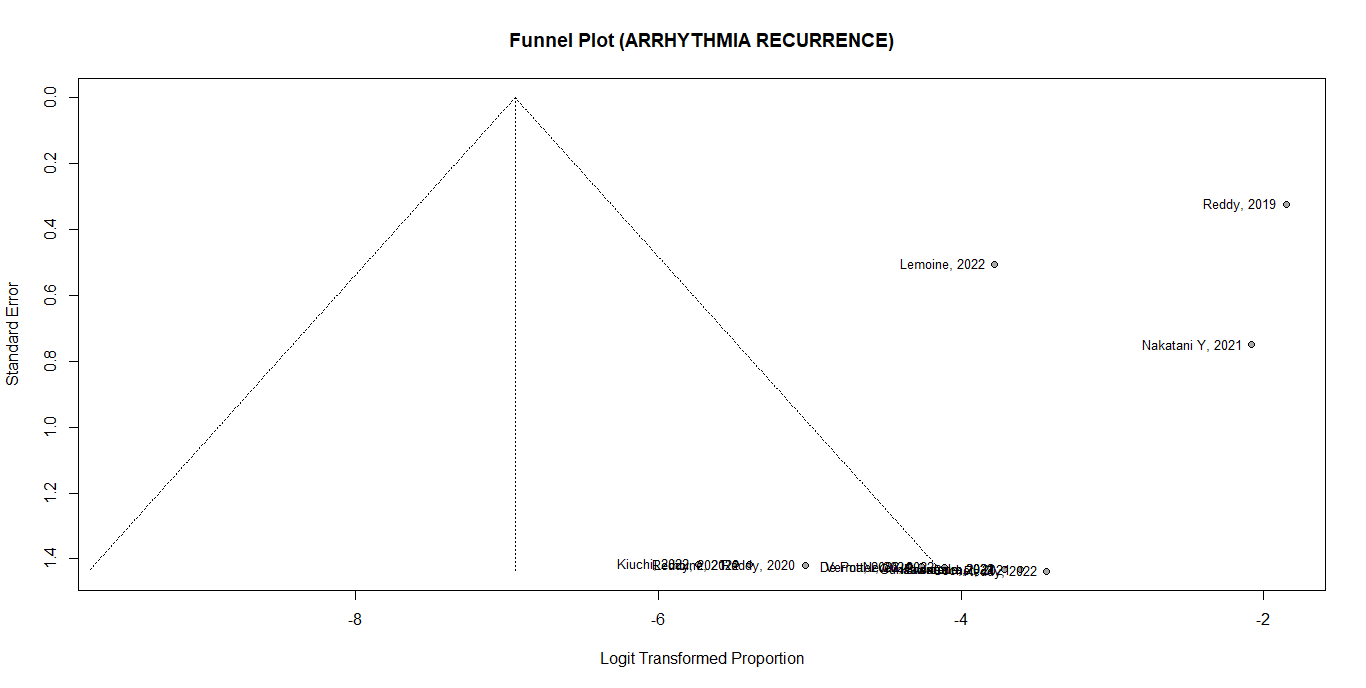


Figure 7Funnel plot for Arrythmia recurrence


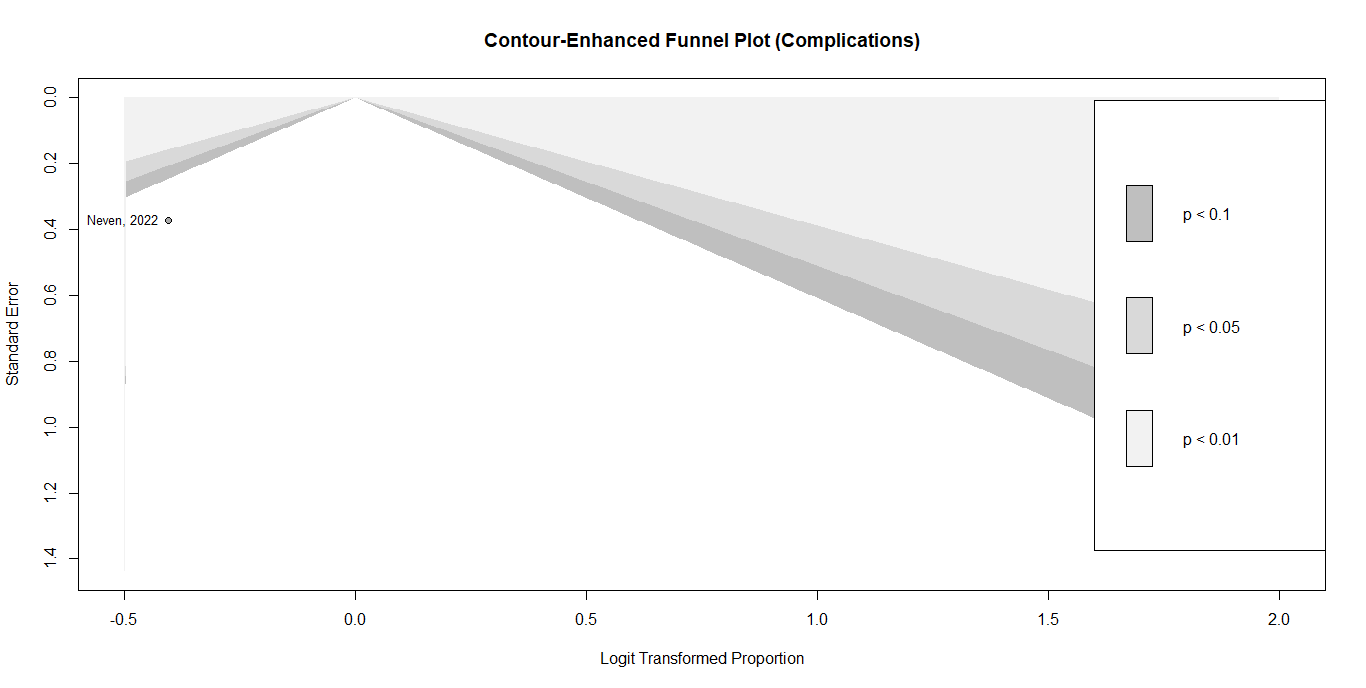


Figure 8Funnel plot for complications

**References**

1. Kawamura I, Neuzil P, Shivamurthy P, et al. How does the level of pulmonary venous isolation compare between pulsed field ablation and thermal energy ablation (radiofrequency, cryo, or laser)? *EP Europace*. 2021;23(11):1757-1766. doi:10.1093/EUROPACE/EUAB150

2. Cochet H, Nakatani Y, Sridi-Cheniti S, et al. Pulsed field ablation selectively spares the oesophagus during pulmonary vein isolation for atrial fibrillation. *Europace*. 2021;23(9):1391. doi:10.1093/EUROPACE/EUAB090

3. Nakatani Y, Sridi-Cheniti S, Cheniti G, et al. Pulsed field ablation prevents chronic atrial fibrotic changes and restrictive mechanics after catheter ablation for atrial fibrillation. *EP Europace*. 2021;23(11):1767-1776. doi:10.1093/EUROPACE/EUAB155

4. Reddy VY, Anter E, Rackauskas G, et al. Lattice-Tip Focal Ablation Catheter That Toggles between Radiofrequency and Pulsed Field Energy to Treat Atrial Fibrillation: A First-in-Human Trial. *Circ Arrhythm Electrophysiol*. 2020; 13:483-495. doi:10.1161/CIRCEP.120.008718

5. Verma A, Boersma L, Haines DE, et al. First-in-Human Experience and Acute Procedural Outcomes Using a Novel Pulsed Field Ablation System: The PULSED AF Pilot Trial. *Circ Arrhythm Electrophysiol*. 2022;15(1): e010168. doi:10.1161/CIRCEP.121.010168

6. Gunawardene MA, Schaeffer BN, Jularic M, et al. Pulsed-field ablation combined with ultrahigh-density mapping in patients undergoing catheter ablation for atrial fibrillation: Practical and electrophysiological considerations. *J Cardiovasc Electrophysiol*. 2022;33(3):345-356. doi:10.1111/JCE.15349

7. Reddy VY, Neuzil P, Koruth JS, et al. Pulsed Field Ablation for Pulmonary Vein Isolation in Atrial Fibrillation. *J Am Coll Cardiol*. 2019;74(3):315-326. doi: 10.1016/J.JACC.2019.04.021

8. Reddy VY, Dukkipati SR, Neuzil P, et al. Pulsed Field Ablation of Paroxysmal Atrial Fibrillation: 1-Year Outcomes of IMPULSE, PEFCAT, and PEFCAT II. *JACC Clin Electrophysiol*. 2021;7(5):614-627. doi: 10.1016/J.JACEP.2021.02.014

9. de Potter T, Reddy V, Neuzil P, et al. Acute safety and performance outcomes from the inspIRE trial using a novel pulsed field ablation system for the treatment of paroxysmal atrial fibrillation. *Eur Heart J*. 2021;42(Supplement_1). doi:10.1093/EURHEARTJ/EHAB724.0380

10. Lemoine MD, Fink T, Mencke C, et al. CA-536-01 PULSED-FIELD ABLATION BASED PULMONARY VEIN ISOLATION: ACUTE SAFETY AND EFFICACY IN A MULTI-CENTER REAL WORLD SCENARIO. *Heart Rhythm*. 2022;19(5): S91. doi: 10.1016/j.hrthm.2022.03.759

11. Reddy VY, Koruth JS, Petru J, et al. PO-711-08 FIRST IN-HUMAN CLINICAL EXPERIENCE WITH PULSED FIELD ABLATION USING A GOLD-TIP FORCE-SENSING 8-FRENCH CATHETER TO TREAT ATRIAL FIBRILLATION: THE PFA-CE STUDY. *Heart Rhythm*. 2022;19(5): S480. doi: 10.1016/j.hrthm.2022.03.1133

12. Neuzil P, Petru J, Skoda J, et al. CA-536-02 PULSED FIELD ABLATION FOR PAROXYSMAL AND PERSISTENT ATRIAL FIBRILLATION USING AN OPTIMIZED BIPHASIC WAVEFORM: REAL CLINICAL DATA FROM SINGLE CENTER. *Heart Rhythm*. 2022;19(5): S91-S92. doi: 10.1016/j.hrthm.2022.03.760

13. Gunawardene M, Schaeffer B, Eickholt C, et al. PO-679-04 PULSED FIELD ABLATION COMBINED WITH ULTRA-HIGH-DENSITY MAPPING IN PATIENTS UNDERGOING CATHETER ABLATION FOR ATRIAL FIBRILLATION. *Heart Rhythm*. 2022;19(5): S354. doi: 10.1016/j.hrthm.2022.03.488

14. Gunawardene M, Lemoine MD, Deneke T, et al. PO-694-05 ACUTE EFFICACY AND SAFETY OF PULSED FIELD ABLATION FOR ATRIAL FIBRILLATION: INITIAL GERMAN MULTICENTER EXPERIENCE. *Heart Rhythm*. 2022;19(5): S411-S412. doi: 10.1016/j.hrthm.2022.03.978

15. Neven K, Füting A, Höwel D, Brokkaar L, Essling A, Reinsch N. PO-634-07 PULSED FIELD ABLATION FOR PAROXYSMAL ATRIAL FIBRILLATION IS SAFE FOR THE BRONCHIAL SYSTEM. *Heart Rhythm*. 2022;19(5): S182. doi: 10.1016/j.hrthm.2022.03.931

16. Kawamura I, Neuzil P, Shivamurthy P, et al. Does pulsed field ablation regress over time? A quantitative temporal analysis of pulmonary vein isolation. *Heart Rhythm*. 2021;18(6):878-884. doi: 10.1016/j.hrthm.2021.02.020
